# Relationship Between Preoperative Anxiety, Salivary Cortisol, Gastric Antrum Volume, and Postoperative Emergence Delirium in Preschool Children Undergoing Adenotonsillectomy

**DOI:** 10.3390/medicina62101945

**Published:** 2026-10-08

**Authors:** Alpay Karaçam, Cihan Döğer, Jan Berkehan Erkilic, Eda Uysal Aydın, Burak Celik, Fazilet Erbay, Bilge Tuncer, Handan Güleç, Ezgi Erkilic

**Affiliations:** 1 Anesthesiology, Ankara Bilkent City Hospital, Ankara 06800, Turkeydredauysal@gmail.com (E.U.A.);; 2 Institut Mondor de Recherche Biomedicale IMRB, Paris-Est Créteil University, 94010 Créteil, France; berkehanerkilic@gmail.com; 3 Faculty of Medicine, Department of Otolaryngology, Head and Neck Surgery, Ankara Yıldırım Beyazıt University, Ankara 06010, Turkey

**Keywords:** perioperative salivary cortisol, PAED, FLACC, mYPAS, pediatric anesthesia, emergence delirium

## Abstract

*Background and Objectives*: This study examined four pre-specified relationships in 47 preschool children (aged 4–7) undergoing adenotonsillectomy: the perioperative change in salivary cortisol in relation to postoperative emergence delirium (PAED) and pain (FLACC) scores, defined a priori as the primary endpoint, and, as secondary, hypothesis-generating endpoints, the associations between preoperative anxiety (mYPAS) and salivary cortisol, between cortisol and gastric antral cross-sectional area (CSA, used as a surrogate for gastric volume), and between intraoperative anesthesia depth (PSI) and PAED scores. *Materials and Methods*: Preoperative anxiety was assessed using the mYPAS scale, followed by salivary cortisol sampling and ultrasonographic measurement of the gastric antral cross-sectional area (CSA, used as a surrogate for gastric volume). Postoperative recovery was monitored using PAED and FLACC scoring systems at 0, 15, and 30 min. *Results*: A moderately strong positive correlation was identified between mYPAS anxiety scores and preoperative salivary cortisol levels. However, no statistically significant correlation was detected between preoperative cortisol and gastric antral CSA. Furthermore, linear mixed-effects modeling did not detect a statistically significant association between the change in cortisol levels and postoperative PAED scores. *Conclusions*: The study confirms that preoperative behavioral anxiety in children is reflected in physiological markers like salivary cortisol. However, no statistically significant association was detected between these cortisol levels and gastric antral CSA, postoperative emergence delirium, or postoperative pain (PAED/FLACC scores) in this cohort. While mYPAS remains a valid tool for assessing preoperative anxiety, our findings do not support cortisol or gastric antral CSA as reliable predictors of post-anesthesia outcomes in this pediatric population; larger studies are needed before concluding that no such relationship exists.

## 1. Introduction

The perioperative period can often be a traumatic experience, particularly for young children undergoing surgery. During this period, three psychological conditions have been described in children: preoperative anxiety, emergence delirium (ED), and newly developed maladaptive behavioral changes after surgery. Anxiety and fear in the preoperative period are particularly important issues that should be carefully considered in children who will undergo anesthesia and surgery [1]. In preschool children, a high level of preoperative anxiety is considered an important risk factor for the development of emergence delirium in the early postoperative period [2]. Anxiety is a complex condition that has multidimensional effects on an individual’s psychological and physiological health. In particular, preoperative anxiety is a critical factor that influences both the success of surgical procedures and the patient’s perioperative experience. Early assessment and appropriate management of this condition in children are of great importance in improving the surgical process and reducing potential long-term negative outcomes. Various scales have been developed to detect preoperative anxiety. Among them, the Modified Yale Preoperative Anxiety Scale (mYPAS) stands out as an effective tool that provides scientific and clinical contributions to the evaluation of preoperative anxiety in children [3].

Stress caused by surgery, anesthesia, and illness leads to an increase in the synthesis and secretion of hormones such as cortisol, glucagon, growth hormone, and catecholamines [4]. This process is regulated by the hypothalamic–pituitary–adrenal (HPA) axis in response to various stimuli, mediated primarily by adrenocorticotropic hormone (ACTH), which stimulates the adrenal cortex. Some molecules, such as cortisol, pass into saliva through passive diffusion, active transport, and ultrafiltration from the blood. Total serum cortisol includes both free (physiologically active) and protein-bound (physiologically inactive) cortisol, whereas salivary cortisol shows a strong correlation with the physiologically active free serum cortisol [5].

Many studies have reported that acute physical stressors reduce gastric emptying rates in healthy adults [6,7,8]. However, previous studies investigating the effect of acute psychological stress on gastric emptying in healthy individuals have reported conflicting results [9,10]. The gastric antrum diameter reflects the anatomical and functional status of the stomach and provides important information for anesthesia management in the perioperative period. The gastric antrum is considered the part of the stomach that provides the most accurate information when assessing gastric contents [11,12]. In a study conducted on 100 fasting children, a linear correlation was identified between the antral cross-sectional area and gastric volume, and this correlation was reported to be stronger when measured in the right lateral decubitus position [13]. Considering that ultrasonography is easy to perform, non-invasive, and cost-effective, it has become an increasingly popular method and has recently begun to be included in clinical guidelines [14,15].

The depth of anesthesia is adjusted according to the type of surgical intervention and the individual requirements of the patient. Achieving an appropriate depth of anesthesia may be particularly challenging in pediatric patients. When the required depth of anesthesia is exceeded, hemodynamic instability and an increased incidence of postoperative delirium may occur [16], whereas a lighter level of anesthesia may create a risk of intraoperative awareness. Monitoring intraoperative electroencephalography (EEG) signals allows visualization of the brain’s response during anesthesia and facilitates titration of anesthetic agents.

Emergence delirium (ED), first described in the 1960s, is a complex psychomotor disturbance occurring in the early period after anesthesia, characterized by inconsolable crying, restlessness, involuntary activity, disorientation, and agitation [17]. Studies in the literature indicate that the incidence of ED is higher in pediatric patients compared with the adult population [18]. The Pediatric Anesthesia Emergence Delirium (PAED) scale is a scoring system designed to observe and accurately evaluate children’s behavior following anesthesia. Proper evaluation of postoperative pain is important when diagnosing ED. Particularly in children who cannot adequately express themselves, distinguishing pain from ED can be difficult. For this purpose, the FLACC (Face, Legs, Activity, Cry, Consolability) scale is used by clinicians to objectively assess pain [19]. The terms “emergence delirium”, “emergence agitation”, and “postoperative agitation” are used variably—at times, interchangeably—in the literature. For clarity and consistency, in this manuscript “emergence delirium (ED)” denotes the categorical diagnosis defined by a PAED score of ≥10, “PAED score” (used as a continuous variable) denotes the underlying construct of behavioral agitation/disorientation on which that diagnosis is based, and “postoperative agitation” is reserved for the general behavioral phenomenon discussed in relation to the literature, independent of the categorical ED threshold.

The primary endpoint of this study was the relationship between the perioperative change in salivary cortisol and postoperative PAED and FLACC scores in preschool-aged children undergoing adenotonsillectomy. The associations between preoperative anxiety (mYPAS) and salivary cortisol, between cortisol and gastric antral CSA, and between intraoperative anesthesia depth (PSI) and PAED scores were evaluated as secondary, hypothesis-generating endpoints. The study also aims to contribute to the development of a multidisciplinary approach to the perioperative management of pediatric patients.

## 2. Materials and Methods

### 2.1. Study Design, Setting, and Participants

This study was conducted in the Ear, Nose and Throat operating room of the Department of Anesthesiology and Reanimation at Ankara City Hospital after obtaining approval from the Ankara City Hospital Medical Research Scientific and Ethics Evaluation Committee No. 2 (decision date: 20 November 2024; meeting decision number: 2-24-107) and written informed consent from the patients’ guardians. After the informed consent forms were explained and approved by the families, 56 patients aged 4–7 years, classified as ASA (American Society of Anesthesiologists) physical status I–II and scheduled for elective adenotonsillectomy, were included in the study. Our study was conducted as a single-center prospective observational clinical study.

Patients whose participation was not approved by themselves or their guardians, those with upper respiratory tract infection, those younger than four years or older than seven years, those with an ASA score of III or higher, those whose surgery started after 11:00 a.m., those using steroids for any reason, those with propofol allergy, and those whose motor and cognitive development were not compatible with their age were excluded from the study. No intravenous or oral premedication was administered to the patients before surgery. Seven patients from whom salivary cortisol samples could not be obtained in the preoperative or postoperative period, or whose samples were insufficient for analysis, and two patients who developed laryngospasm or bronchospasm were excluded from the study, yielding a final analytic sample of 47 children.

### 2.2. Study Procedures

First, age, height, body weight, body mass index, sex, ASA classification, and the presence of any systemic diseases were recorded. Subsequently, the first baseline salivary cortisol sample was collected in the preoperative area. During salivary cortisol collection, care was taken to ensure that at least 60 min had passed since tooth brushing or any oral intake of solid or liquid food.

While the patients were in the preoperative waiting area with their families, the Modified Yale Preoperative Anxiety Scale (mYPAS) was applied. This scale includes observational measurements of anxiety in five categories: activity, vocalization, emotional expressivity, state of apparent arousal, and interaction with family. Scores range from 23.3 to 100, with higher scores indicating greater anxiety. The evaluation results were recorded. No pharmacological sedative or anxiolytic premedication was administered to any patient prior to the mYPAS assessment or baseline salivary cortisol sampling, so that both measures reflected each child’s unmedicated preoperative anxiety and stress state.

Afterward, the patients were positioned at 45° semi-recumbent and in the right lateral decubitus (RLD) position. The Butterfly iQ (Butterfly Network, Inc., Guilford, CT, USA), a portable ultrasound device whose probe depth and frequency can be optimized between 1 and 5 MHz, was used to obtain cross-sectional images of the gastric antrum; the device has preset settings within its application that allow it to simulate either a convex or linear probe [20]. In the right lateral decubitus position, the gastric antrum was identified according to the standard protocol [12] in the sagittal or slightly oblique sagittal plane in the epigastrium, along the edge of the left lobe of the liver and anterior to the aorta or inferior vena cava. Oblique images were avoided to ensure accurate acquisition of the transverse cross-section of the antrum.

The cross-sectional area (CSA) of the gastric antrum was measured assuming an elliptical shape using the formula CSA = π × AP × CC/4 [21], where AP and CC represent the anteroposterior and craniocaudal diameters of the gastric antrum, respectively. To ensure accurate measurement of the antral area, all images were obtained while the antrum was at rest, between peristaltic contractions. The mean value of three CSA measurements obtained in the right lateral decubitus position was recorded. All gastric ultrasound examinations were performed by the same researcher who conducted the other perioperative assessments in this study; a formal inter- or intra-observer reproducibility analysis (e.g., an intraclass correlation coefficient) was not performed, and we acknowledge this as a methodological limitation (Section 4). Because the antral CSA—rather than a directly converted volumetric quantity—is the parameter that was actually measured, this CSA value is what is referred to as “gastric volume” elsewhere in this manuscript, consistent with its established use as a validated ultrasonographic surrogate for gastric content/volume in the literature [11,12,13].

When the patient was transferred to the operating room, routine ASA monitoring (5-lead ECG, non-invasive blood pressure monitoring, and pulse oximetry) was applied. After cleaning the forehead skin with an alcohol swab, the SedLine probe was placed on the patient’s forehead. This procedure was performed before anesthesia induction in cooperative patients and immediately after induction in patients who required sedation. In all patients, a SedLine monitor (Root; Masimo Corporation, Irvine, CA, USA) with pediatric sensors was used. Patient State Index (PSI) values were recorded at 10-min intervals from anesthesia induction until the patient was deemed suitable for transfer from the operating room to the recovery room. The minimum PSI value reached during surgery was also recorded.

During peripheral venous catheterization, a blood sample was obtained for serum cortisol analysis and sent to the laboratory. Following the recording of preoperative parameters (demographic data, ASA physical status, and gastric antrum diameter measurements), peripheral venous access was established, and anesthesia was induced with propofol. Neuromuscular blockade was achieved with 0.6 mg/kg rocuronium, and patients were intubated with an appropriately sized endotracheal tube. Additionally, fentanyl at a dose of 1 µg/kg was administered during induction.

For anesthesia maintenance, inhalational anesthesia with sevoflurane at approximately 1 minimum alveolar concentration (MAC) was administered in a mixture of 50% oxygen and 50% air, along with remifentanil infusion, while considering PSI levels and hemodynamic parameters. All patients routinely received intravenous proton pump inhibitor (1 mg/kg) and intravenous methylprednisolone (1 mg/kg) before surgery. Postoperative analgesia was provided with paracetamol (15 mg/kg). In children with insufficient analgesia, ibuprofen (20 mg/kg) was planned as rescue analgesia.

Upon arrival in the recovery room, emergence delirium was assessed using the Pediatric Anesthesia Emergence Delirium (PAED) scale. The first measurement was recorded at minute 0, followed by subsequent measurements at 15 and 30 min. Patients were evaluated based on eye contact, purposeful movements, awareness of surroundings, general restlessness, and consolability. Each parameter was scored between 0 and 4, and the total PAED score was calculated accordingly. The assessments were performed by the same researcher and conducted in an environment minimally affected by external factors. A PAED ≥ 10 score was used a priori as the diagnostic threshold for the categorical diagnosis of emergence delirium, consistent with previously validated cutoffs described in the original PAED validation study [22]. PAED scores were used in two complementary ways in this study: as a continuous outcome variable in the linear mixed-effects models described in Section 2.5, and, after applying the ≥10 threshold, as a categorical variable to determine the overall incidence of emergence delirium (4.25% in this cohort; see Section 3.1).

In all observations, care was taken to ensure that pain was not misinterpreted as delirium. For this purpose, the FLACC (Face, Legs, Activity, Cry, Consolability) scale was used. Pain assessment using the pediatric FLACC scale was performed by the same researcher simultaneously with PAED measurements at 0, 15, and 30 min after the patient arrived in the recovery room. Children who were asleep were considered not to be experiencing pain. The FLACC score was calculated as the sum of the evaluated parameters, and scores greater than 4 were considered indicative of the need for additional analgesia.

Postoperative salivary cortisol samples were obtained again in accordance with the same procedures used for preoperative saliva sample collection. No patient required reoperation due to postoperative bleeding, and all surgeries were performed by the same ear, nose, and throat surgical teams. Adenotonsillectomy was performed using the coblation technique in all patients, and the surgical technique did not differ between patients included in the final analysis.

### 2.3. Salivary Cortisol Analysis Protocol

Salivette^®^ cortisol collection tubes (Sarstedt, Nümbrecht, Germany) were used for saliva sampling. After explaining the procedure clearly to the child, the swab contained in the Salivette kit was chewed for at least 2 min. The first collected sample was sent to the laboratory within 30 min. Saliva samples were centrifuged (1000× *g*, 2 min, 4 °C) to remove cellular debris, and the supernatant was obtained as a clear fluid. The samples were stored at −20 °C until analysis.

Salivary cortisol levels were analyzed using the electrochemiluminescence immunoassay (ECLIA) method with the Cobas e 411 analyzer (Roche Diagnostics GmbH, Mannheim, Germany). The assay operates based on a competitive principle. After the first and second incubation periods, the resulting reaction mixture was aspirated into the measuring cell, and microparticles were magnetically captured onto the electrode surface. Application of voltage to the electrode induced chemiluminescent emission, which was measured using a photomultiplier. The results were determined by the analyzer using a specifically generated two-point calibration along with a master calibration curve provided via the reagent barcode or e-barcode.

### 2.4. Sample Size and Power Analysis

Based on the study by Kara et al. [23], the minimum required sample size for evaluating the change in salivary cortisol levels before and after anesthesia was calculated to be at least 45 patients, assuming a statistical power of 95% and a type I error rate of 0.05. The calculation was performed using the open-source software R (version 3.1.7).

Additionally, based on the study by Kawana et al. [24], which investigated children aged 3–6 years undergoing minor surgical procedures and evaluated anxiety levels using the Visual Analog Scale (VAS) in relation to gastric volume, the effect size for assessing the relationship between anxiety and gastric volume was calculated as 0.484. With a statistical power of 95% (α = 0.05), the required sample size was again calculated to be at least 45 patients using R (version 3.1.7).

Considering a potential dropout or exclusion rate of approximately 25%, the final required sample size was determined to be 56 patients. The two independent power calculations, both yielding a minimum requirement of 45 patients, correspond to the two pre-specified endpoints of this study (the cortisol–PAED/FLACC relationship and the anxiety–gastric CSA relationship, respectively); because both calculations converged on the same minimum, a single target sample size was adopted for the study as a whole rather than requiring separate cohorts. The 56-patient enrollment target already incorporated an anticipated 25% attrition. In the event, 9 of the 56 enrolled patients (16.1%) were excluded (7 owing to inadequate salivary cortisol sampling and 2 owing to laryngospasm or bronchospasm), an attrition rate lower than the 25% originally anticipated, yielding a final analytic sample of 47 children. This final sample size still exceeded the minimum of 45 patients required by both a priori power calculations.

### 2.5. Statistical Analysis

Data analysis was performed using RStudio (version 2024.09.0+375). During the evaluation of the study data, descriptive statistical methods were used, including frequency, percentage, minimum, maximum, mean, standard deviation, median, and interquartile range (IQR). The normality of the data distribution was assessed using the Shapiro–Wilk test and, when necessary, the Kolmogorov–Smirnov test, as well as graphical methods (histogram, Q–Q plot, stem-and-leaf plot, and boxplot).

To examine differences between groups, the independent samples *t*-test was used for normally distributed continuous variables, whereas the Mann–Whitney U test was used for variables that did not follow a normal distribution. For comparisons of categorical variables, the chi-square test or Fisher’s exact test was applied. Repeated measures were analyzed using repeated measures ANOVA or the Wilcoxon test. Relationships between variables were examined using Pearson’s or Spearman’s correlation tests. Independent-samples tests were used only for between-group comparisons involving different individuals (e.g., comparisons between sexes); within-subject comparisons of the same biomarker measured at different perioperative time points in the same patients (e.g., preoperative versus postoperative salivary cortisol) were instead analyzed using the paired-samples *t*-test or, when normality assumptions were not met, the Wilcoxon signed-rank test, consistent with the repeated-measures approach described above. Throughout the Results, correlation coefficients and, where available, 95% confidence intervals are reported alongside *p*-values so that the magnitude and precision of each association, rather than statistical significance alone, can be judged; for the linear mixed-effects models, effect direction and *p*-values are reported in text, with full model outputs (coefficients and 95% confidence intervals for each fixed effect) available from the corresponding author upon request.

In this study, linear mixed-effects models were used for advanced statistical analysis. Initially, simple univariate models were applied, and subsequently the models were expanded by adding additional variables based on clinical relevance. These additional variables were selected according to their clinical significance and Akaike Information Criterion (AIC) values. The models included PAED and FLACC scores as dependent variables. Time and the rate of cortisol change were defined as fixed effects, while individual intercepts were included as random effects to account for variability between individuals. This approach allowed accurate evaluation of both group-level and individual-level outcomes while considering repeated measurements over time and inter-individual differences.

The PAED model was structured to evaluate the interaction between time and the rate of cortisol change. The FLACC model was designed with a similar structure to assess postoperative pain scores. In both models, additional covariates—including age, sex, body mass index (BMI), ASA score, duration of surgery, gastric antral CSA, and PSI score—were included as fixed effects. This modeling structure enabled comprehensive analysis of both the individual and combined effects of independent variables on PAED and FLACC scores. Given the sample size of 47 participants relative to the number of covariates included (up to eight fixed effects per model), we recognize the potential for model overfitting and reduced precision of individual coefficient estimates. Covariate selection was therefore restricted to variables with an established or biologically plausible link to PAED/FLACC outcomes, and further guided by Akaike Information Criterion (AIC)-based comparison to avoid unnecessary model complexity; this trade-off, and the residual risk of instability in detecting modest effect sizes, is discussed further in the Limitations.

## 3. Results

### 3.1. Descriptive Analyses

A total of 47 children were included in the study. The mean age of the participants was 5.83 ± 1.05 years, and 70.2% were male. The effects of preoperative and postoperative cortisol levels and gastric antral CSA on PAED and FLACC scores were analyzed.

The mean preoperative salivary cortisol level was 0.37 ± 0.41 µg/dL (median: 0.28; range: 0.12–2.95), whereas the mean postoperative salivary cortisol level was 2.02 ± 0.99 µg/dL (median: 1.98; range: 0.32–3.88).

The demographic and clinical characteristics of the study population are presented in Table 1.

Preoperative serum and salivary cortisol levels, postoperative salivary cortisol levels, preoperative gastric antral CSA, percentage change in salivary cortisol, and PSI values are summarized in Table 2.

Postoperative PAED and FLACC scores at different time points are shown in Table 3.

The mean percentage change in salivary cortisol levels between the preoperative and postoperative periods was 7.45 ± 4.89, with a median of 6.62 (range: 0.424–19.1).

Postoperative FLACC scores were 6.94 ± 1.63 at 0 min, 3.45 ± 0.90 at 15 min, and 1.15 ± 0.91 at 30 min. Similarly, PAED scores were 14.7 ± 2.72 at 0 min, 8.09 ± 2.45 at 15 min, and 5.26 ± 9.15 at 30 min.

Variance equality between sexes was assessed using F-tests to determine the appropriate type of *t*-test. Equal variances were observed for PAED at 0 min, PAED at 15 min, FLACC at 0 min, and FLACC at 15 min (*p* > 0.05), and therefore independent samples *t*-tests assuming equal variances were applied. However, for PAED at 30 min, variances were not equal (*p* < 0.001), and thus the Welch *t*-test was used.

No statistically significant differences between sexes were observed for PAED scores at 0 min (*p* = 0.406), 15 min (*p* = 0.682), or 30 min (*p* = 0.155). Similarly, FLACC scores at 0 min (*p* = 0.864) and 15 min (*p* = 0.929) did not differ significantly between sexes. However, a significant difference was found for FLACC scores at 30 min (*p* < 0.001), with higher mean scores in males (1.42) compared with females (0.50).

### 3.2. Correlation Analyses

**Figure 1:**
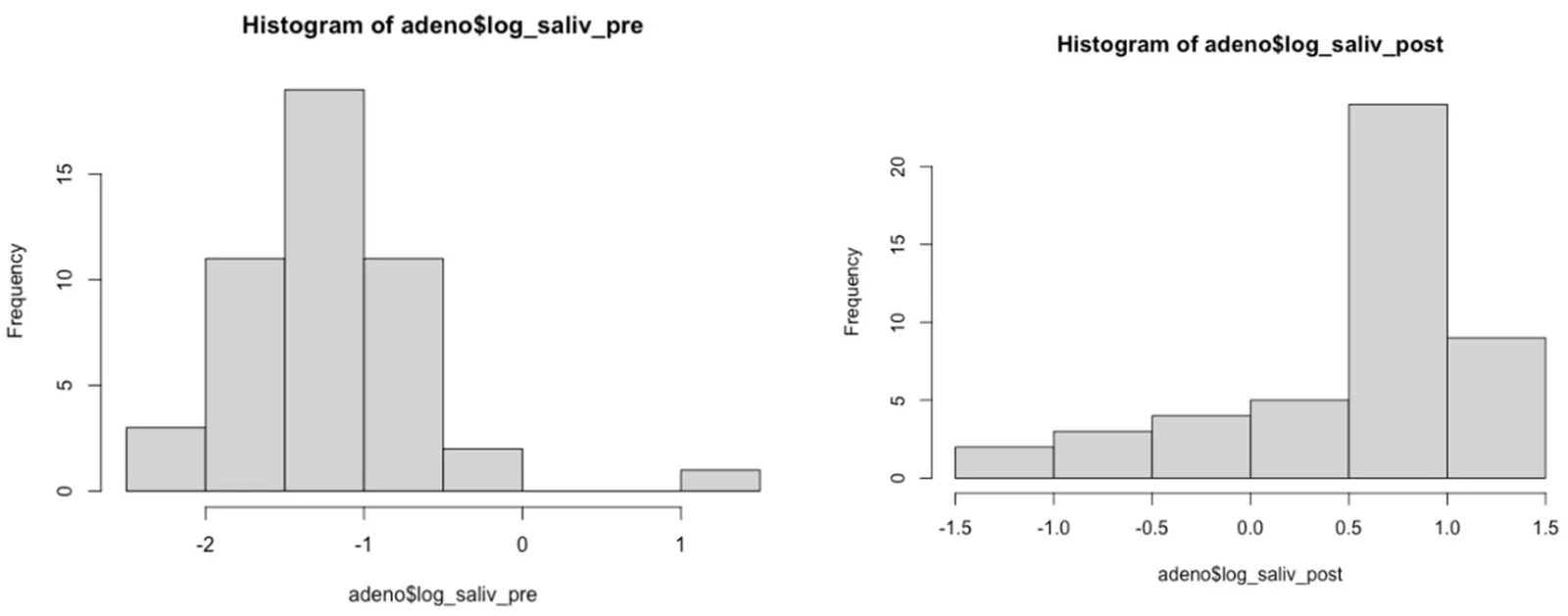
Logarithmic distributions of salivary cortisol levels (preoperative, (**left**); postoperative, (**right**)).

The logarithmically transformed distributions of cortisol levels clearly demonstrate the distribution patterns of preoperative and postoperative cortisol concentrations (Figure 1).

Scatter plots illustrating the relationship between preoperative salivary cortisol levels and postoperative PAED and FLACC scores showed no clear linear relationship at any of the three postoperative time points.

The graphical representation shows a moderately strong positive relationship between preoperative salivary cortisol levels and mYPAS scores, whereas the relationship between postoperative cortisol levels and mYPAS scores was weak and not statistically significant. The scatter plots indicate that higher preoperative cortisol levels tend to be associated with higher mYPAS scores, while postoperative cortisol levels did not demonstrate a noticeable association with mYPAS scores.

A significant positive correlation was found between preoperative salivary cortisol levels and mYPAS scores (Pearson’s correlation, r = 0.50; *p* < 0.001; 95% CI: 0.25–0.69).

A weak negative correlation was observed between preoperative salivary cortisol levels and gastric antral CSA, which did not follow a normal distribution; however, this relationship was not statistically significant (Spearman’s ρ = −0.20; *p* = 0.178). Similarly, no significant relationship was found between preoperative serum cortisol levels and gastric antral CSA (ρ = 0.05; *p* = 0.759).

The correlation matrix was analyzed to evaluate the relationship between preoperative stress levels and postoperative agitation and pain scores. The correlation between preoperative cortisol levels and PAED scores at 0 min was 0.34, indicating a mild positive relationship between stress levels and agitation. However, this relationship gradually decreased over time, declining to 0.20 at 15 min and 0.07 at 30 min, suggesting that the effect of preoperative stress diminishes during the postoperative period.

Correlations between FLACC scores and preoperative cortisol levels were substantially weaker. At the first pain assessment, the correlation was 0.08, which is not considered clinically meaningful. This correlation decreased to 0.05 at 15 min and −0.12 at 30 min, indicating that preoperative stress did not have a substantial influence on postoperative pain scores.

Correlations between PAED scores across time points were generally strong. A correlation of 0.56 was observed between the first and second measurements, and 0.57 between the second and third measurements, suggesting temporal consistency in agitation scores, although the magnitude of agitation decreased over time, possibly reflecting the influence of external factors such as sedation or clinical interventions.

Similarly, pain scores demonstrated relatively strong correlations across time points: 0.52 between the first and second measurements and 0.56 between the second and third measurements, indicating temporal consistency in pain assessments.

When the relationship between agitation and pain scores was examined, a high correlation of 0.71 was observed at the initial measurement. However, this relationship gradually decreased over time, declining to 0.30 at 30 min.

### 3.3. Mixed-Effects Regression Analyses

In the linear mixed-effects model evaluating PAED scores, the rate of cortisol change was examined as an independent variable and adjusted for other covariates including time, gastric antral CSA, PSI score, age, sex, BMI, ASA score, and duration of surgery. The analysis showed no significant effect of cortisol change on PAED scores (*p* = 0.755), indicating that a one-unit increase in cortisol change was not associated with a significant change in PAED scores.

Similarly, gastric antral CSA did not show a statistically significant association with PAED scores within the same model (*p* = 0.977). After adjustment for other covariates, no statistically significant association was detected between gastric antral CSA and PAED scores. Given the sample size, this should be interpreted as an absence of detectable association in this cohort rather than evidence that no true association exists. The PSI score was also not a significant predictor of PAED scores (*p* = 0.287); changes in PSI were not associated with a statistically significant variation in postoperative delirium scores after adjustment for other variables.

In the linear mixed-effects model evaluating FLACC scores, no statistically significant association was detected between cortisol change rate and FLACC scores (*p* = 0.419). Given the modest sample size, this finding should be interpreted as an absence of a detectable association rather than proof that cortisol change has no true relationship with postoperative pain scores. Similarly, gastric antral CSA was not significantly associated with FLACC scores (*p* = 0.934), and the PSI score also did not demonstrate a statistically significant association with FLACC scores (*p* = 0.779); neither finding should be read as establishing that these variables have no influence on postoperative pain.

Linear mixed-effects models constructed using preoperative serum cortisol levels to evaluate PAED and FLACC scores also revealed no significant effects of preoperative serum cortisol levels on either outcome (*p* = 0.195 and *p* = 0.196, respectively). For the PAED model, variations in preoperative serum cortisol levels were not associated with postoperative delirium; similarly, in the FLACC model, serum cortisol levels did not demonstrate a significant influence on postoperative pain scores.

## 4. Discussion

In this study, we found that preoperative and postoperative salivary and serum cortisol levels, as well as gastric antral CSA measurements, had a limited role in predicting postoperative PAED and FLACC scores, whereas the correlation observed between preoperative salivary cortisol levels and preoperative anxiety scores indicates that salivary cortisol may still hold value as a biomarker for evaluating perioperative anxiety. Nevertheless, our findings suggest that the clinical utility of salivary cortisol in the assessment of preoperative anxiety should be further clarified through randomized controlled trials conducted in different patient groups.

The physiological stress response is mediated primarily through the hypothalamic–pituitary–adrenal (HPA) axis, a complex neuroendocrine network whose final product, cortisol, has been widely used as a biological indicator of stress and has been the focus of numerous investigations [25,26,27]. Salivary cortisol levels correlate strongly with free serum cortisol concentrations, making salivary measurements a practical and non-invasive marker of stress sensitivity [23,28]. Several studies in both pediatric and adult populations support an association between salivary cortisol and anxiety [23], and some reports suggest that salivary cortisol may serve as a predictor of perioperative neurocognitive outcomes [29]. Because salivary protein secretion is largely under sympathetic control, changes in saliva composition may also serve as sensitive indicators of stress arising from the sympathetic-adrenomedullary limb of the stress response [30].

In our study, we identified a moderate positive correlation between preoperative salivary cortisol levels and mYPAS scores, supporting the hypothesis that salivary cortisol may reflect preoperative anxiety in pediatric patients. Similar findings were reported by Kara et al., who demonstrated a positive relationship between serum and salivary cortisol levels and mYPAS scores in both the preoperative and postoperative periods among 81 patients aged 5–17 years undergoing circumcision [31]. Hsu et al., in a study of 110 children aged 1 month to 17 years undergoing sedation, reported that sedation and anesthesia caused a significant increase in salivary cortisol levels regardless of sedation level or procedure performed [32]. Likewise, Silveira et al. reported a positive association between salivary cortisol levels and mYPAS scores in 46 children aged 5–12 years, in whom the group with higher anxiety levels was, interestingly, the group accompanied by parents [33]. In contrast, Wennström et al., in a study of 93 children undergoing day-case surgery, found no relationship between salivary cortisol and preoperative anxiety levels [34]. Patil et al., evaluating 20 healthy children aged 4–8 years around their first dental examination, reported no correlation between salivary cortisol and Corah anxiety scores [35], and Kanegane et al., in 73 adult patients requiring emergency dental intervention, found no relationship between salivary cortisol and anxiety or sex, suggesting instead that cortisol levels might be higher in patients experiencing pain [36]. Such discrepancies across studies may be explained by differences in patient populations, surgical or procedural context, and anxiety assessment methods. The mYPAS itself is frequently used in preschool- and school-aged children given its high sensitivity (85%) and specificity (92%) [3].

In our study, only preoperative salivary cortisol was significantly associated with anxiety scores, whereas no correlation was found for postoperative salivary cortisol or preoperative serum cortisol. Evaluation of the graphs comparing PAED with preoperative salivary cortisol levels showed that salivary cortisol values were concentrated within a narrower range in the preoperative period, whereas they displayed wider variation postoperatively. This finding suggests a relatively similar basal stress state among individuals preoperatively, whereas the postoperative distribution may reflect individual differences in the response to surgical stress.

Regarding cortisol and postoperative neurocognitive outcomes, Ma et al., in 119 adult patients with acute coronary syndrome and renal dysfunction, demonstrated that patients who developed postoperative delirium had higher serum cortisol levels at admission, before percutaneous coronary intervention, than those who did not [37]. Han et al., in 120 adults undergoing non-cardiac surgery, found a significant association between diurnal changes in preoperative salivary cortisol and postoperative cognitive dysfunction [29]. Unlike these studies, we did not detect a statistically significant relationship between the percentage change in salivary cortisol levels and postoperative PAED scores, nor did preoperative serum cortisol predict PAED or FLACC scores in our mixed-effects models.

With respect to gastric volume, acute stress has been reported to inhibit gastric emptying in experimental animal models and in human studies—initially in rats by Taché et al. [38] and later in eight healthy adult volunteers by Lee et al., who also found no significant relationship between auditory stress and salivary cortisol levels [39]. However, clinical studies evaluating preoperative anxiety have generally failed to demonstrate a clear relationship between anxiety and gastric volume: other reports have shown that preoperative anxiety does not prolong gastric emptying in adults or children and has no effect on gastric acidity or volume [40,41], and Hong et al., in 96 women aged 16–60 years undergoing gynecological surgery, found no relationship between anxiety (assessed by visual analog scale) and gastric volume [42]. Our findings are consistent with these results, as we did not observe a significant correlation between preoperative cortisol levels (salivary or serum) and gastric antral CSA.

The depth of anesthesia has also been proposed as a potential factor influencing emergence delirium. Faulk et al., monitoring anesthesia depth with the bispectral index (BIS) in 400 patients aged 1–12 years undergoing dental procedures, reported that the duration of deep anesthesia had no effect on emergence agitation or postoperative behavioral changes [43]. Similarly, Frederick et al. found no relationship between BIS-guided anesthesia depth and emergence agitation in 40 children aged 2–8 years undergoing ophthalmic surgery [44], and Larsen et al. reported that Narcotrend monitoring in 96 patients aged 1–6 years undergoing day-case surgery did not reduce the rate of emergence agitation [45], consistent with our findings. In a retrospective cohort of 66 patients aged 1–9 years undergoing day-case surgery lasting less than 2 h, Kanaya et al. showed that mean PSI during general anesthesia and preoperative anxiety might be associated with PAED scores during recovery [46]; PSI values have also been shown to be comparable to BIS values during the perioperative period [47]. In our study, we used the minimum intraoperative PSI value to evaluate its relationship with PAED scores; ultimately, minimum PSI was not a significant predictor, and a one-unit increase in PSI did not produce a measurable effect on PAED scores after adjustment for other variables.

Patients in our cohort experienced their highest PAED scores within the first 15 min in the recovery room, consistent with previous studies regarding the timing of peak emergence delirium after surgery [48,49]. Liu et al., using data from 4974 patients aged 1–18 years, reported an ED incidence of 1.3% after adenoidectomy and tonsillectomy, with PAED scores significantly higher in male participants [50]. In the broader literature, the incidence of ED in the pediatric population has been reported over a wide range, from 2% to 80% [51,52,53], a variability likely explained by differences in study design, assessment scales, and the surgical and anesthetic procedures examined. In our study, despite the relatively small sample size, delirium was detected in only two male patients (4.25%), a comparatively low rate.

Regarding sex differences, Karišik et al., in 60 children aged 3–6 years undergoing unilateral inguinal hernia repair, found that boys had a greater postoperative analgesic requirement than girls [54]. In our study, analyses by sex showed no significant differences in PAED and FLACC scores at most time points; however, FLACC scores at 30 min were significantly higher in boys than in girls, suggesting a subtle, time-dependent sex difference in pain perception whose overall clinical impact appears limited. Separately, Tadesse et al., in a prospective cohort of 208 adults undergoing elective surgery, reported that preoperative anxiety was associated with greater postoperative pain, as evidenced by higher tramadol consumption in more anxious patients [55]. In our study, however, the linear mixed-effects model evaluating FLACC scores did not demonstrate a significant relationship between the salivary cortisol change ratio and FLACC scores, and the corresponding correlations from 0 to 30 min first decreased and then became negative.

Taken together, our correlation and scatter-plot analyses point to a consistent pattern: the association between preoperative stress markers and postoperative agitation/pain was weak and diminished further over the recovery period, whereas the association between agitation and pain scores was initially strong (r = 0.71) but likewise weakened by 30 min (r = 0.30). This pattern suggests that early postoperative agitation may be driven substantially by inadequately controlled pain rather than by preoperative stress biomarkers, and that effective early analgesia may be a more actionable lever for reducing agitation than preoperative stress-marker screening.

An important question raised by our findings is why preoperative anxiety, gastric antral CSA, and salivary cortisol levels were not associated with postoperative emergence delirium in this cohort. Several explanations may account for this dissociation. First, pediatric emergence delirium—unlike adult postoperative delirium—is thought to arise primarily from the pharmacodynamic properties of volatile anesthetic agents, particularly sevoflurane, which produce rapid recovery of motor function relative to cortical and sensory reorientation in an immature central nervous system; this agent-driven, transient dissociation between motor and cognitive recovery may overwhelm any modest contribution from circulating stress hormones or baseline anxiety. Second, the hypothalamic–pituitary–adrenal (HPA) axis in preschool children is still maturing, and cortisol reactivity to psychological stress may be blunted or highly variable at this age, weakening its value as a biomarker in this population. Third, the factors more consistently associated with emergence delirium in the pediatric literature include younger age, use of rapid-onset/rapid-offset anesthetic agents, ENT or ophthalmic surgery, difficult temperament, inadequately controlled postoperative pain, and the rapidity of emergence itself, rather than a single circulating biomarker—consistent with our own finding that early agitation correlated more strongly with pain scores than with preoperative cortisol. Finally, unlike pediatric emergence delirium, postoperative delirium in adults is more strongly linked to neuroinflammation, cerebral hypoperfusion, polypharmacy, and cumulative comorbidity burden—mechanisms in which cortisol plausibly plays a more direct pathophysiological role—which may explain why associations established in adult cohorts did not translate to our pediatric cohort.

A major strength of this study is that prospectively collected data allowed salivary and serum cortisol levels and gastric antral CSA measurements to be evaluated together within the same cohort, providing a comprehensive, multidimensional perspective on the biological and physiological dimensions of surgical stress and addressing a gap in the literature by simultaneously examining the hormonal stress response alongside gastric antral CSA. Several limitations should nonetheless be acknowledged. Cortisol sampling and measurement procedures, as well as device sensitivity, may be prone to error, and postoperative variables in particular may be affected by biological and environmental factors. The relatively limited sample size may also have reduced the ability to detect some significant associations, particularly those with borderline *p*-values; larger, ideally multicenter, studies are needed to further clarify the role of cortisol and other biomarkers in predicting postoperative behavioral outcomes in children. Additional limitations include the single-center design, which may limit the generalizability of our findings to other institutional settings and anesthetic practices; the modest sample size relative to the number of covariates included in the mixed-effects models, which raises the possibility of model overfitting and reduced power to detect modest associations, as noted in Section 2.5; potential measurement variability arising from single-operator ultrasound assessment without formal inter- or intra-observer reliability testing (Section 2.2); and the fact that all patients underwent a single surgical procedure (adenotonsillectomy), which limits the generalizability of our findings to children undergoing other types of surgery.

## 5. Conclusions

In this study, we concluded that preoperative salivary and serum cortisol levels were not correlated with gastric antral CSA, and that these parameters played a limited role in explaining postoperative PAED and FLACC scores. However, a significant correlation was observed between preoperative anxiety scores and preoperative salivary cortisol levels.

These findings suggest that biological responses to surgical stress alone may not be sufficient to explain subjective pain and agitation scores in the postoperative period. Postoperative pain and agitation appear to be influenced by individual and time-dependent factors, highlighting the multifactorial nature of perioperative stress responses.

Future studies should include larger patient populations and different surgical procedures and further explore multidisciplinary assessment approaches. In particular, randomized controlled trials are needed to better understand the impact of preoperative stress management strategies on postoperative outcomes.

Salivary cortisol may serve as a potential non-invasive biomarker for assessing preoperative anxiety in pediatric patients, although its role in predicting postoperative behavioral outcomes appears limited.

## Figures and Tables

**Table 1:** Demographic and clinical characteristics of the study population.

Variable	*n*	%	Mean (SD)	Median (Min–Max)
Age (years)	47	100	5.83 (1.05)	6.00 (4.00–7.00)
Sex				
Female	14	29.8		
Male	33	70.2		
Weight (kg)	47	100	22.8 (6.43)	21.0 (14.0–45.0)
Height (cm)	47	100	117 (9.26)	118 (93.0–137.0)
ASA Score				
ASA I	42	89.4		
ASA II	5	10.6		

**Table 2:** Preoperative and postoperative cortisol, gastric antral CSA, and PSI values.

Variable	Mean (SD)	Median (Min–Max)
Preoperative serum cortisol	15.5 (6.6)	15.8 (5.0–30.3)
Preoperative salivary cortisol	0.370 (0.411)	0.284 (0.121–2.95)
Postoperative salivary cortisol	2.02 (0.990)	1.98 (0.320–3.88)
Pre-post salivary cortisol change	7.45 (4.89)	6.62 (0.424–19.1)
Preoperative gastric volume	2.7 (0.99)	2.5 (1.7–4.9)
Patient State Index (PSI)	37 (4.6)	39 (27–44)

**Table 3:** Postoperative PAED and FLACC scores at 0, 15, and 30 min.

Variable	Mean (SD)	Median (Min–Max)
PAED Score		
0 min	14.7 (2.72)	15.0 (9.0–20.0)
15 min	8.09 (2.45)	8.0 (4.0–16.0)
30 min	5.26 (0.915)	4.0 (1.0–6.0)
FLACC Score		
0 min	6.94 (1.63)	7.0 (4.0–10.0)
15 min	3.45 (0.904)	3.0 (2.0–6.0)
30 min	1.15 (0.908)	1.0 (0–4)

## Data Availability

Data are available from the corresponding author upon reasonable request.
